# Ultrasound-Induced Amino Acid-Based Hydrogels With Superior Mechanical Strength for Controllable Long-Term Release of Anti-Cercariae Drug

**DOI:** 10.3389/fbioe.2021.703582

**Published:** 2021-10-18

**Authors:** Liying Ling, Lei Zhu, Yibao Li, Chunhua Liu, Linxiu Cheng

**Affiliations:** ^1^Jiangxi Key Laboratory of Organo-Pharmaceutical Chemistry, Chemistry and Chemical Engineering College, Gannan Normal University, Ganzhou, China; ^2^Research Center for Environmental Engineering and Technology, School of Geography and Environmental Engineering, Gannan Normal University, Ganzhou, China

**Keywords:** ultrasound-induced, amino acid-based, hydrogels, long-term release, anti-cercariae

## Abstract

Stimulus-responsive hydrogels are significantly programmable materials that show potential applications in the field of biomedicine and the environment. Ultrasound as a stimulus can induce the formation of hydrogels, which exhibit the superior performance of different structures. In this study, we reported an ultrasound-induced supramolecular hydrogel based on aspartic acid derivative *N*,*N*′-diaspartate-3,4,9,10-perylene tetracarboxylic acid imide, showing superior performance in drug release. The results show that the driving force of this ultrasonic induced hydrogel could be attributed to hydrogen bonding and π-π interaction. The rheological and cytotoxicity test illustrate excellent mechanical properties and biocompatibility of the hydrogel. The anti-*Schistosoma japonicum* cercariae (CC) drug release results show large drug loadings (500 mg/ml) and long-term release (15 days) of this hydrogel. This study demonstrates that this hydrogel may serve as a slow-release platform for anti-CC.

## Introduction

Supramolecular hydrogels with various functions have been paid much attention, and a rapidly growing number of discoveries have been reported (Li et al., 2006) in recent years. Compared with the traditional polymer hydrogel, supramolecular hydrogels are a novel class of cross-linked soft materials *via* self-assembly (Wang et al., 2010, 2021; Yu et al., 2020) driven by various non-covalent interactions. These driving forces such as hydrogen bonding, metal–ligand coordination, host–guest recognition, and electrostatic interaction remarkably reduce the structural flexibility and alter the macroscopic performance, resulting in the formation of three-dimensional (3D) cross-linked networks (Appel et al., 2010; Ma et al., 2012; Tian et al., 2013; Zhang et al., 2016). Particularly, the gelation and drug loading of supramolecular hydrogels could be achieved simultaneously in an aqueous environment and without covalent cross-linking, which has been widely used in biomedicine, tissue engineering, biosensor, drug delivery, and other research areas (Díaz et al., 2011; Chakraborty et al., 2012).

Unfortunately, the strengths of supramolecular hydrogels are generally lower than those of polymer gels because their formation is based on weak non-covalent interactions. The practical application of supramolecular gels has been highly restricted. Researchers have been attempted to increase the strength of supramolecular gels, but it always impaired their original flexibility (Loos et al., 1997; Draper et al., 2015; Cheng et al., 2018). A general strategy to increase the strength of supramolecular gels would be quite useful.

Non-Covalent forces readily respond to external stimuli, such as biological targets, pH, anions, small molecule chemicals, and redox behavior. So many biotechnological applications based on supramolecular hydrogels are the important research area (Segarra-Maset et al., 2013; Adhikari and Kraatz, 2014; Afrasiabi and Kraatz, 2015). Hydrogen bonding, as the most common non-covalent force, plays an important role in the process of supramolecular hydrogel assembly. Tang et al. (2010) reported a high-strength hydrogel which was prepared by using the super-strong hydrogen bonding between diaminotriazine. This hydrogel could be applied in the field of cell transfection and separation. Amino acid and the related derivatives are not only desired biomolecules but also receptors and donors of hydrogen bonding. Amino acid derivative-based hydrogels have been actively studied for applications in various research fields (Langhals, 2005; Zhao et al., 2007; Li and Wonneberger, 2012). Strong hydrogen bonding could improve the mechanical properties of the supramolecular gel. Dai et al. (2015) designed a novel hydrogelator—a polymer compound equipped with amino acid residues at side chains. The hydrogel-based compound shows high mechanical strength, stability, and thermoplasticity. These interesting properties could be attributed to double hydrogen bonding from carboxyl groups of amino acid residues. Mechanical strength is an important property of supramolecular gel. In the field of drug release, mechanical strength plays a decisive role in the release rate of drug molecules. Sutton et al. (2009) suggested that the release rate does not depend on the size of the drug molecule but on the mechanical strength of the supramolecular gel carrier.

Schistosomiasis is a zoonotic parasitic disease caused by schistosomiasis infection. This kind of disease causes serious harm to human health and the social economy of epidemic areas (Njoroge et al., 2014; Bergquist and Gray, 2019; Wu et al., 2021; Zhou et al., 2021). Up to date, more than 250 million people worldwide and nearly 800 million people are at risk of schistosomiasis infection. The schistosome life cycle is complex, of which the aquatic cercaria (CC) stage is the only infectious stage. We invented a highly effective insecticide for anti-CC, which can diffuse on the water surface (Li et al., 2013). In a natural environment, the anti-cercarial results of the effective insecticide have not been ideal because the pesticides quickly lose their effectiveness in the water soon after they are applied. So, we reported some drug carrier materials based on a supramolecular gel to solve this problem, and it still needs to be improved to extending release time for practical application.

Thus, a supramolecular single-component hydrogel based on amino acid derivatives was constructed via self-assembled behavior. It was expected to have excellent mechanical properties to extending release time, good drug release properties, and anti-cercarial ability.

## Experiment

### Materials

All reagents were purchased from commercial suppliers and used as received without further purification. *N*,*N*′-diaspartic acid-3,4,9,10-tetracarboxylic diimide (NAAPD) has been synthesized and characterized as the method we reported. Niclosamide derivatives (NMD) were synthesized and characterized in a laboratory.

### Characterization

The FT-IR spectra were carried out using KBr disks on an AVATAR 360 FT-IR spectrophotometer (Nicolet, Wisconsin, USA) at room temperature. The morphology of hydrogels was observed by a scanning electron microscope 98 (FEI, QUANTA 450, Hillsboro, USA) at 20 kV. The rheological properties of hydrogels were measured using a HAAKE RheoStress 6000 rheometer (Offenburg, Germany). The storage (G′) and loss (G″) moduli as functions of time were monitored at 25°C at a frequency of 1.0 Hz. To investigate the mechanical properties and flow behavior of hydrogels, frequency sweeps from 0.1 to 100 rad s^−1^, and a steady shear test from 0.01 to 10 s^−1^ were performed. Confocal laser scanning microscope (CLSM) images were taken with CLSM FV 1000 invert microscope (OLYMPUS) under 100× magnification at an excitation wavelength of 488 nm.

### Formation of Hydrogel

The mixture solvent of 1.0 ml H_2_O and tetrahydrofuran (THF) was added to the sample bottle with a certain mass of NAAPD to obtain a suspension. The suspension was fully dissolved by ultrasonography until the sample was gelatinized by the solution and turned into a fluid-free semisolid. The gelation was confirmed by inverting the tube with no flowing liquid.

### Cytotoxicity Test

The HeLa cells were seeded in 96-well plates with a density of 10^5^ cells per well and then incubated overnight in Dulbecco's modified Eagle medium (DMEM) containing 10% fetal bovine serum. The cells were washed with phosphate-buffered saline (PBS) and then incubated in a fresh medium containing NAAPD/NMD (10, 20, 50, and 100 mM) for 24 h. The media were washed with PBS, then the cells were incubated in DMEM containing tetrazolium dye (MTT) (0.25 mg/ml), and cultured for 4 h at 37°C. After disposed of, the plates were shaken for 5 min and then analyzed with a SpectraMax M5 plate reader to record the absorbance at 490 nm.

### Drug Loading Test

A total of 1.0 ml NAAPD solutions were configured in H_2_O/THF, and different qualities of the NMD were added in the complex solutions. The compounds were sustained with ultrasonic treatment for 2 h at room temperature. The formation of the gels was observed to investigate the maximum drug loadings of this kind of supramolecular gel material.

### *In vitro* Release

The 1.0 ml hydrogel sample with 5% ω/ν of NAAPD, which was loaded with 20.0 mg of NMD, was dried via freeze-drying in a vacuum for 6 h. The drug release research was performed by immersing the xerogel sample in a 200.0-ml portion of water at 25°C. A total of 0.5 ml of the solution was taken out from the glass bottle at intervals, and equal fresh water was then added to ensure the same total solution volume. The concentrations of the NMD were measured by the quantitative determination method at 326.5 nm.

### Anti-Cercarial Activity Experiments

According to the *in vitro* release test, a 300-μl solution was removed from the glass in 96-well plates at different release times. *Schistosoma japonicum* CC were acquired from the water surface in a conical flask after the infected *Oncomelania hupensis* was soaked with bright lighting for 2 h. The CC were removed from the plates, including the above solution, at different release times. The activity of the CC was observed every 30 min via biological microscopy.

## Results and Discussion

**Figure 1:**
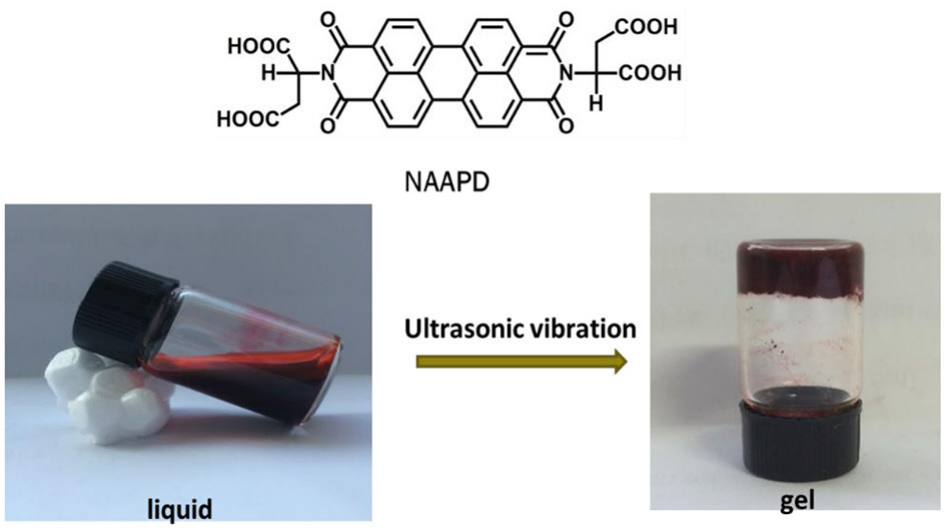
Chemical structures of *N*,*N*′-diaspartic acid-3,4,9,10-tetracarboxylic diimide (NAAPD), and preparation diagram of supramolecular hydrogels.

We tested the gelation ability of NAAPD in a mixed solution (THF/H_2_O = 1:1) in a typical test-tube experiment. It was found that a rufous and steady gel is generated under ultrasound conditions for 2 h at 25°C. The minimum gelator concentration is 2.0% ω/v. As shown in Figure 1, the chemical structure of *N*,*N*′-diaspartic acid-3,4,9,10-tetracarboxylic diimide (NAAPD) indicates that this amphipathic molecule is equipped with a hydrophobic core and hydrophilic side chains. It is a perfect gelator to form network structures because of π-π stacking and remarkable donors and receptors of hydrogen bonds.

**Figure 2:**
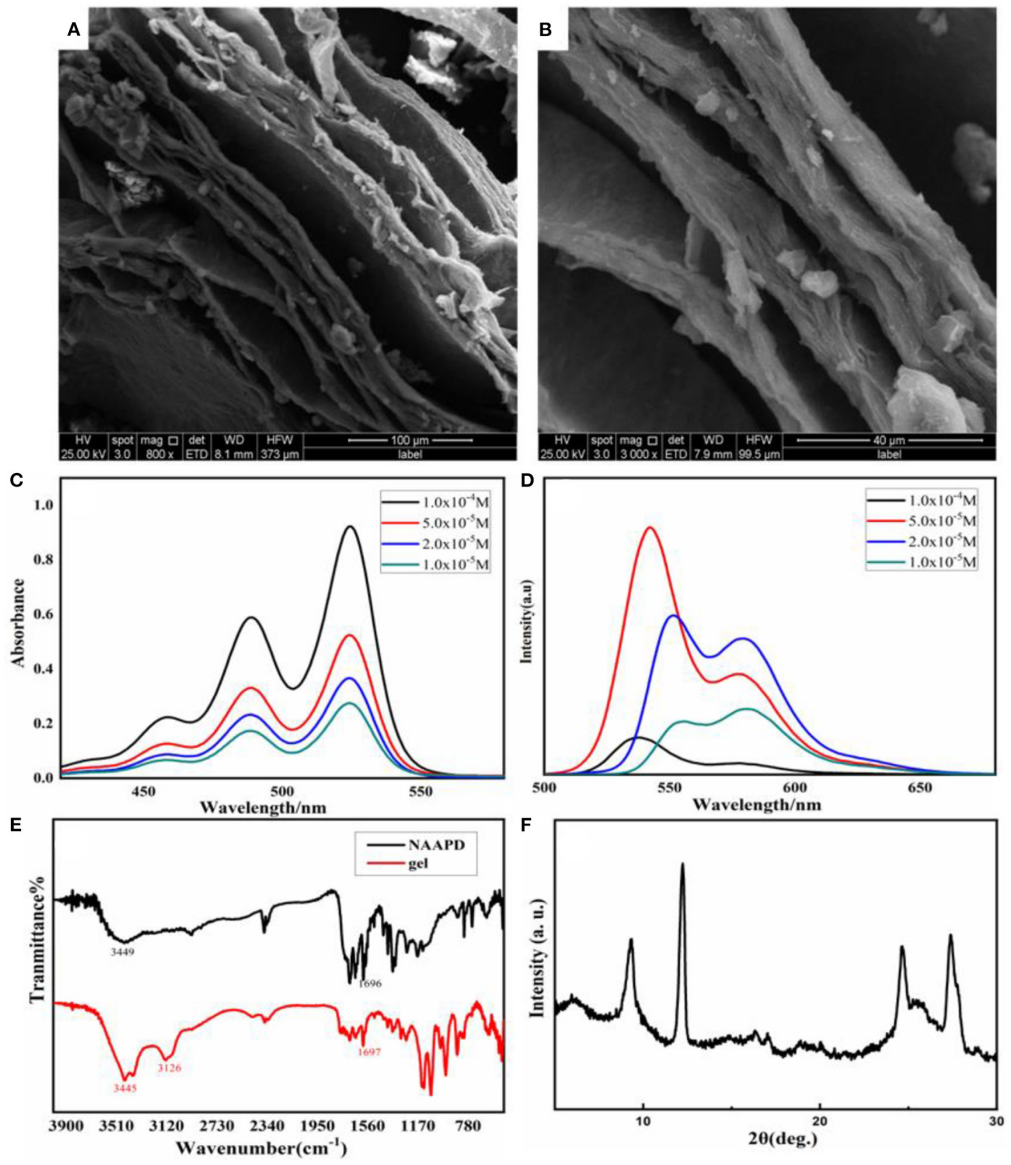
Scanning electron microscopy (SEM) images of the NAAPD xerogel: **(A)** in larger scale and **(B)** in small scale. Spectra of NAAPD solutions in THF/H_2_O = 1:1 with different concentrations: **(C)** UV-vis spectra of the NAAPD loose gel at room temperature (path length = 2.0 mm), **(D)** fluorescence spectra of the solution and the gel of the NAAPD system at room temperature, at λ_ex_ = 486 nm (path length = 5.0 mm); **(E)** FT-IR spectra diluted with KBr for a xerogel (black) and powder (red); and **(F)** experimental powder XRD pattern of NAAPD xerogel.

Micro-topography of gel was observed by scanning electron microscopy (SEM) to investigate self-assembly information. In Figure 2, the gel is composed of manifested lamellar structure. These lamellar structures are sprawling stacked to form many pores in the cross-stacking process. These pores could not only capture the solvent to generate a physical gel but also allow the hydrogel to load the drug.

UV-vis spectra, fluorescence spectra, infrared spectra, and X-ray diffraction (XRD) were recorded to explore the driving force of the gel system. UV-vis spectra (Figure 2C) show significant absorption at 489 and 524 nm at the concentration of 1.0 × 10^−5^ M. With increasing concentration, the peak intensity of UV absorption increases. This could be attributed to the almost identical stacking geometry of the aromatic systems between the different concentrations of NAAPD and the aggregation of the NAAPD gel system (Li Y. B. et al., 2014; Li et al., 2015). The fluorescence spectra of NAAPD solutions in THF/H_2_O = 1:1 with different concentrations are shown in Figure 2D. The maximum emission wavelength of NAAPD is at 551 nm, and the second-largest emission wavelength is at 579 nm. As the concentration increases, the intensity of the fluorescence emission peaks increases. However, as the concentration of NAAPD reaches 10^−4^ M, the intensity decreases and the wavelength shift reduces significantly. According to previous studies (Bullock et al., 2009; Malinovskii et al., 2010; Würthner et al., 2011), perylene diimide derivatives emit enhanced fluorescence in dilute solution, while fluorescence decreases or even quenching in aggregation state. This could be attributed to the aggregation induced by π-π electronic coupling, which results in a diminution of fluorescence emission intensity (Li Y. B. et al., 2014; Li et al., 2015). As shown in Figure 2E, the single band of pure NAAPD at 1,696 cm^−1^ correspond to hydrogen bonding C=O vibration, which is similar to NAAPD gel with the single band at 1,697 cm^−1^. It is speculated that both pure NAAPD and NAAPD gel could form hydrogen bonding interactions (Zhang et al., 2012; Li et al., 2014). The UV-vis spectra, fluorescence spectra, and infrared spectra imply that π-π stacking and hydrogen bonding between NAAPD make great contributions to the formation of the gel.

Powder X-ray diffraction patterns were performed using freeze-dried samples of NAAPD hydrogel. As shown in Figure 2F, the small-angle region shows irregular signals at 8.9°, 12.3°, 24.6°, and 27.2°, corresponding to *d* spacing values of 9.6, 7.2, 3.6, and 3.2 Å, respectively. This result indicates that NAAPD gel is a polycrystalline structure (Tripathi et al., 2014; Oveshnikov et al., 2020).

Rheological measurements for NAAPD hydrogels (5% ω/ν) were carried out to investigate the dynamic mechanical properties of the gel. In Supplementary Figure 1a, with a frequency of 6.28 rad/s, the stress scanning results show that the samples are in a gel state in the beginning. With G′ and G″ intersection, NAAPD begins to show the flowing dynamics, indicating the typical physical gel of NAAPD hydrogels. From a frequency sweep experiment (Supplementary Figure 1b), it could be observed that the dynamic storage modulus G′ is larger than the corresponding dynamic loss modulus G″. Notably, the value of dynamic storage modulus G′ almost reaches 40,000 Pa, showing the high mechanical strength of the gel. With the increase of frequency, only G′ and G″ slightly increase with no mutation, indicating small frequency response and better mechanical properties of the gel. As shown in Supplementary Figure 1c, a time sweep at a strain of 30% displays that viscoelasticity maintains invariability over 6,000 s. The above results suggest that this extremely stable hydrogel has excellent mechanical properties.

**Figure 3:**
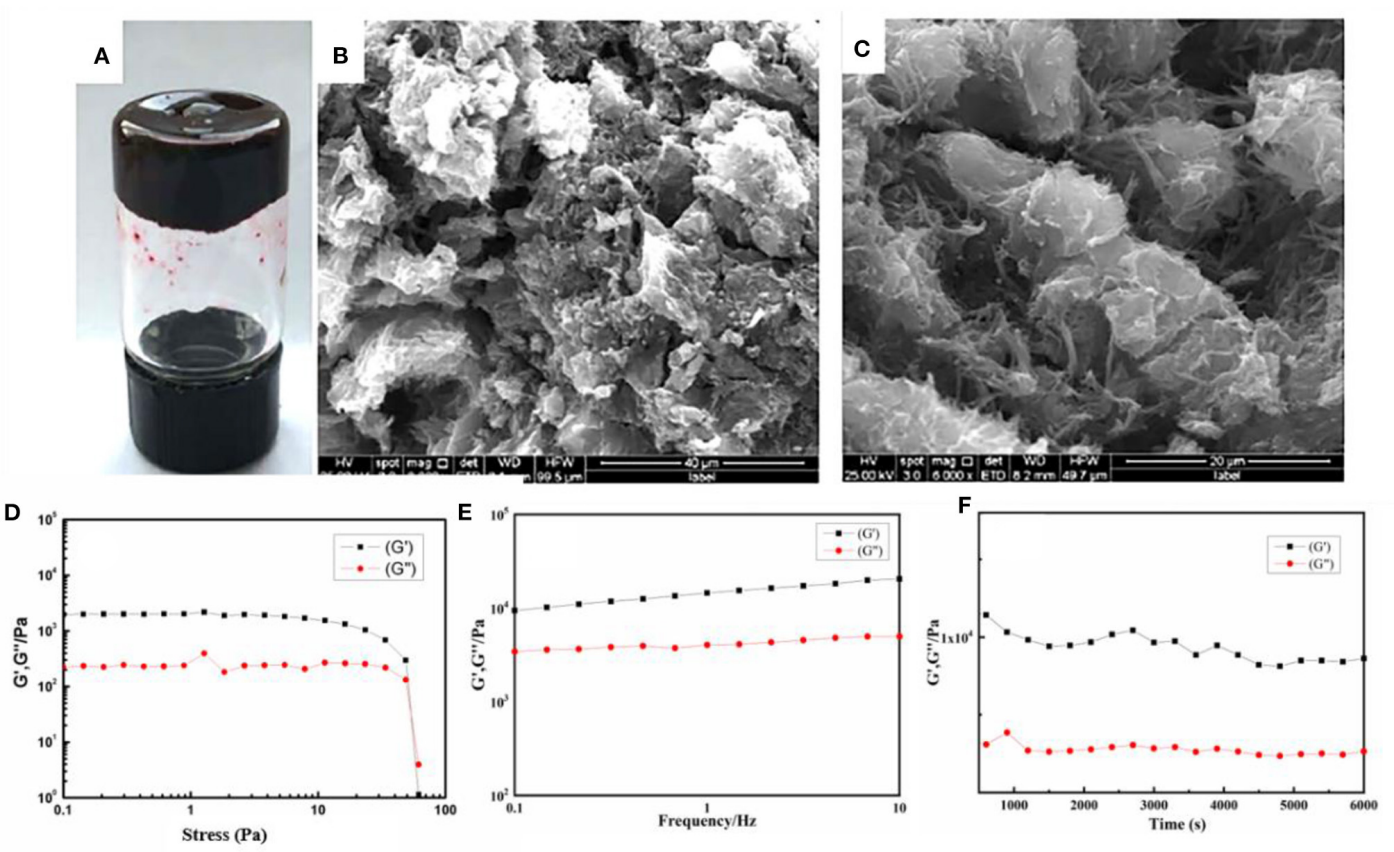
**(A)** Photo of the NAAPD–niclosamide derivatives (NMD)-loaded gel, SEM images of the samples **(B)** for NAAPD drug-loaded in larger scale, **(C)** for NAAPD–NMD-loaded gel in small scale, **(D)** strain sweep for NAAPD–NMD-loaded gel at 5% w/v, **(E)** frequency sweep of the NAAPD–NMD-loaded gel at the strain of 0.1%, and **(F)** time sweep of the NAAPD–NMD-loaded gel at the strain of 30%.

Drug content is an important parameter to evaluate the properties of the drug-loaded materials. As shown in Figure 3A, 1.0 ml NAAPD gel (5% w/v) could load a maximum of 500 mg of NMD drug without destroying gel formation. The SEM images (Figures 3B,C) show that the drugs are loaded into the intrinsic 3D porous network structure and that blocky structures instead of lamellar structures could be found in the network structure. It could be speculated that the NMD molecule has been loaded into the hydrogel and formed a stable structure.

To explore whether NMD has successfully loaded NAAPD gel, UV-vis spectra and fluorescence spectra were recorded at various concentrations. UV-vis spectra (Supplementary Figure 2) show significant absorption at 532, 548, and 594 nm at the concentration of 1.0 × 10^−5^ M. The peak intensity of UV absorption increases when the concentration increases to 2.0 × 10^−5^ M. As the concentration reaches 5.0 × 10^−5^ and 1.0 × 10^−4^ M, the UV absorption decreases. This could also be attributed to the similar stacking geometry of the aromatic systems between the different concentrations of the NAAPD–NMD system and the aggregation state of the NAAPD–NMD system. The fluorescence spectra of NAAPD–NMD solutions with different concentrations are displayed in Supplementary Figure 3. The maximum emission wavelength of pure NMD is 324 nm. As the concentration increases, the emission wavelength at 533 nm (NAAPD) remains unchanged, while the peak 324 nm (NMD) shows a red-shift (~6 nm for 1.0 × 10^−5^ M) comparing pure NMD. It is speculated that the aggregation induced by π-π electronic coupling between NAAPD molecules, NAAPD, and NMD molecules.

Furthermore, the rheology experiments were performed to explore the stability of NAAPD drug-loaded gel. As shown in Figure 3D, the value of storage modulus G′ is higher than the corresponding dynamic loss modulus G″ in the strain sweep, which suggests that this hydrogel is a viscoelastic material. According to Figure 3E, G′ is invariably larger than G″ from 0.01 to 10 Hz, and their difference remains unchanged, which means that this gel is insensitive to an external force. The time sweep exhibits that the viscoelasticity of hydrogels does not change in 6,000 s as shown in Figure 3F. This result suggests that drug molecules are completely wrapped in the hydrogel and that the stable hydrogel system has good mechanical properties.

**Figure 4:**
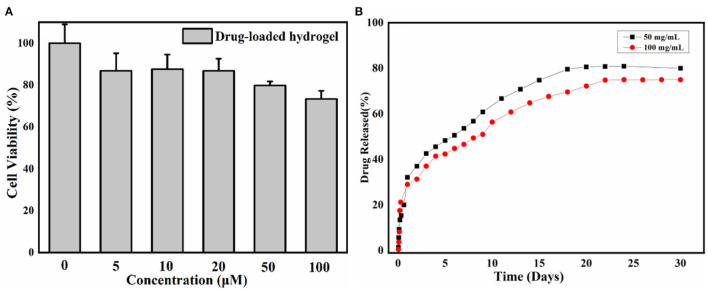
**(A)** The 24 h cell viability test of NAAPD drug-loaded gel in different concentrations and **(B)** drug release behavior of NMD from the NAAPD drug-loaded gel at 25°C in water.

Furthermore, an MTT assay was utilized to evaluate the cytotoxicity of the gel on HeLa cells to explore the biocompatibility of the gelator. A cytotoxicity diagram is obtained after 24 h incubation of HeLa cells with various amounts of hydrogel (0, 10, 20, 50, and 100 mM) at different ratios. Based on the results of the MTT assay shown in Figure 4A, after being incubated with hydrogels at 10 and 20 mM, cell viability remains above 80%, while incubated with hydrogels at 50 and 100 mM, cell viability remains about 80%, implying low toxicity of the hydrogels. These results illustrate that NAAPD drug-loaded gel has good biocompatibility.

The release profiles of NMD from the NAAPD–NMD-loaded with different quality drug loadings were carried out at 25°C in water (Figure 4B). In the first 24 h, the sustained release rate of the drug is fast. Two days later, the sustained release rate decreases gradually with the release rate becoming quite low after 25 days.

It could be observed that about 80% of NMD is released from the hydrogel over 30 days. The amount of NMD drugs has a weak effect on the sustained release behavior of the NAAPD gel system. The release amount of NMD for the NAAPD drug-loaded system (100 mg/ml) is much less than that of the NAAPD drug-loaded system (50 mg/ml), showing a faster release rate of the drug with a lower concentration. It is speculated that the NMD molecule contains an amide group and a hydroxyl group which could participate in the assembly of the gel because NMD could interact with the NAAPD molecule to form hydrogen bonding. The higher the concentration of NMD, the more stable the gel is and less likely to be destroyed, hence the release rate decreases as the concentration of NMD increases.

Furthermore, the anti-cercarial ability of the release system was investigated. The anti-cercarial activity investigation was conducted in a released solution at different release time. As shown in Table 1, the effect of anti-cercarial activity is prominent. First, CC were incubated in the solution for 1 h, and the mortality rate of CC is nearly 0 (incubation time: 30 min). However, CC inactivation starts after 60 min of incubation, and the mortality rate reaches 87.5% (incubation time: 180 min). Furthermore, the mortality rate of CC reaches 100% after incubated with the solution released from the gel (release time: 24 h) for 60 min. Therefore, the NAAPD–NMD-loaded hydrogel release system maintains commendable bioactivity against CC in the water environment.

**Table 1:** The mortality rate of *Schistosoma japonicum* cercariae for the hydrogel release system *N*,*N*′-diaspartic acid-3,4,9,10-tetracarboxylic diimide–niclosamide derivative-loaded gel (25°C).

**Release time (h)**	**Mortality (%)**			
	**30 min**	**60 min**	**120 min**	**180 min**
1	0.00	6.25	31.25	87.5
2	2.44	51.22	75.61	100
5	22.22	81.48	92.59	100
24	50.00	100	100	100
200	76.92	100	100	100
Blank	0.00	0	0	0

## Conclusion

In this study, ultrasound-induced supramolecular hydrogel based on aspartic acid derivatives is successfully fabricated. Various characterizations reveal that the driving forces are the hydrogen bonding between carboxyl and the π-π interaction between the core. The hydrogel exhibits excellent mechanical strength and biocompatibility. Furthermore, NAAPD supramolecular hydrogel displays a large drug loading ability with the drug NMD that could connect with NAAPD via hydrogen bonding. The release time of NMD is about 15–22 days with the release rate remaining about 80%. Moreover, the results of the anti-cercarial ability demonstrate good efficiency of the release system in killing cercariae in a water environment. This ultrasound-induced supramolecular hydrogel with excellent mechanical strength may serve as a slow-release platform for anti-cercariae.

## Data Availability Statement

The original contributions presented in the study are included in the article/Supplementary Material, further inquiries can be directed to the corresponding author/s.

## Author Contributions

LL, LZ, and CL carried out the experiments. LC wrote the manuscript. YL revised the manuscript. All authors contributed to the article and approved the submitted version.

## Funding

This study was supported by the National Natural Science Foundation of China (Grant Numbers: 52060002 and 21962003).

## Conflict of Interest

The authors declare that the research was conducted in the absence of any commercial or financial relationships that could be construed as a potential conflict of interest.

## Publisher's Note

All claims expressed in this article are solely those of the authors and do not necessarily represent those of their affiliated organizations, or those of the publisher, the editors and the reviewers. Any product that may be evaluated in this article, or claim that may be made by its manufacturer, is not guaranteed or endorsed by the publisher.
