# Supplementary material for: Ultrasound-Induced Amino Acid-Based Hydrogels With Superior Mechanical Strength for Controllable Long-Term Release of Anti-Cercariae Drug
Source: Front Bioeng Biotechnol. 2021 Oct 18;9:703582. doi: 10.3389/fbioe.2021.703582 (PMC8558479; doi:10.3389/fbioe.2021.703582)
Supplement: Supplementary file 1 [file Data_Sheet_1.docx]

## Figures


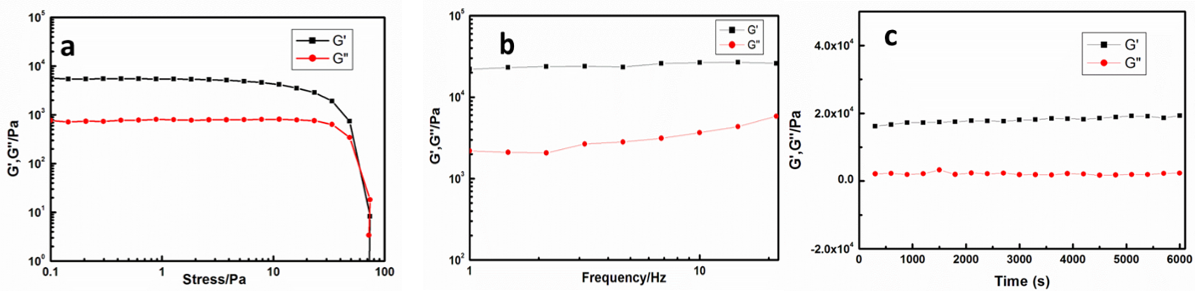


**Supplementary Figure 1.** (a) strain sweep for NAAPD gel at 5% w/v concentration; (c) frequency sweep of the NAAPD gel at the strain of 0.1%; (d) time sweep of the NAAPD gel at the strain of 30%.


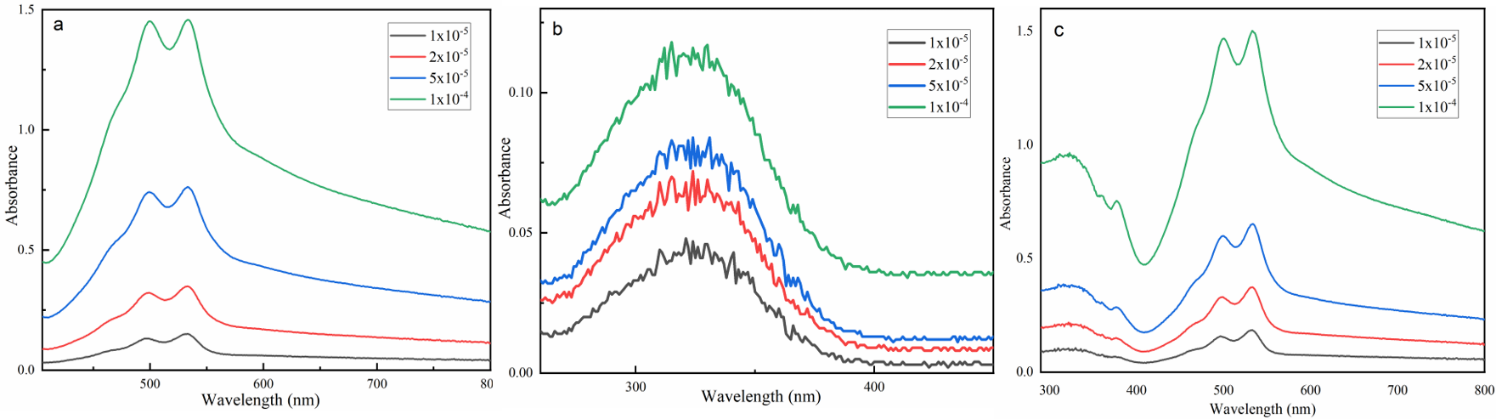


**Supplementary Figure 2.** UV-Vis spectra of (a) the NAAPD loose gel, (b) NMD, (c) NAAPD-NMD system at room temperature [path length = 2.0 mm].


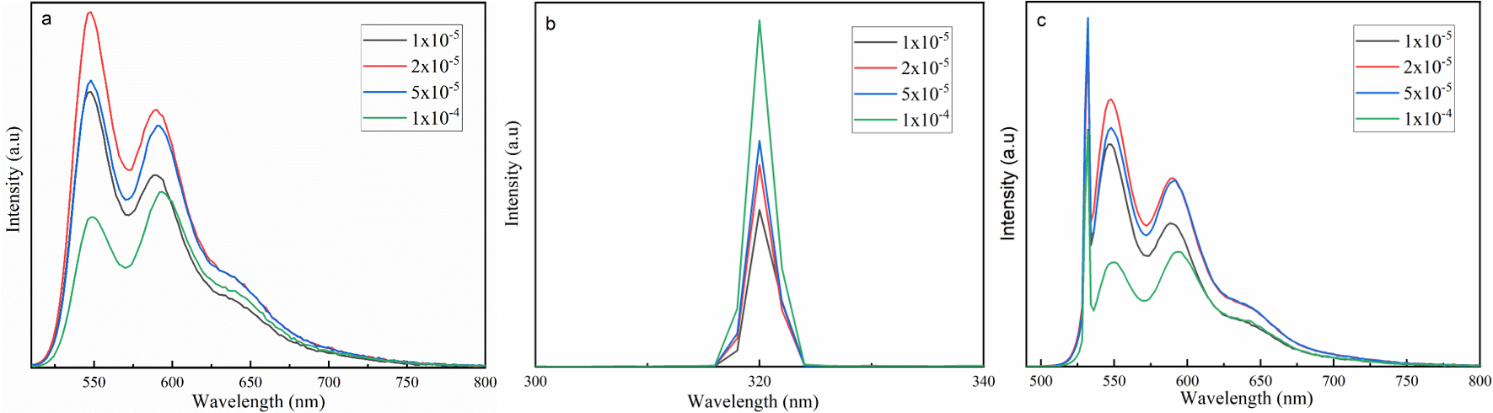


**Supplementary Figure 3.** Fluorescence spectra of (a) the NAAPD loose gel, (b) NMD, (c) NAAPD-NMD system at room temperature, at λ_ex_ = 486 nm [path length = 5.0 mm]
